# Refining the employability of university students from low-income families: a qualitative study on the influential factors and mechanism of employability

**DOI:** 10.3389/fpsyg.2026.1718245

**Published:** 2026-03-02

**Authors:** Dan Wang, Bohan Liu, Yujie Duan, Yue Liang, Di Wu, Dan Zhang, Chao Song

**Affiliations:** 1 College of Education Science, Jilin Normal University, Siping, Jilin, China; 2 Institute of Education, University College London, London, United Kingdom; 3 School of Psychology, Nanjing Normal University, Nanjing, Jiangsu, China

**Keywords:** employability, low-income families, psychological capital, social cognitive career theory, university students

## Abstract

**Introduction:**

Graduate employability is increasingly emphasized in higher education, yet how students from low-income families develop employability remains under-theorized, particularly regarding the interplay between psychological resources and contextual constraints.

**Methods:**

Using reflexive thematic analysis, we analyzed semi-structured interviews with 11 university students from low-income families to examine perceived influences and mechanisms underlying employability development.

**Results:**

Nineteen sub-themes converged into four higher-order themes: psychological capital, resource-compensatory proactivity, goal clarity, and capability enactment. Self-efficacy and optimism energized proactive behaviors that sharpened career goals. Goal clarity then prompted deliberate practice that strengthened learning and self-management, resilience, and socio-communicative skills. These processes unfolded within family and university contexts, including economic constraint, prestige-based stratification, teachers’ guidance, seniors’ experience transfer, extracurricular participation, and internship and part-time work, which operated as filters or scaffolds. Mastery experiences further fed back to reinforce self-efficacy and optimism.

**Conclusion:**

The resulting conceptual model localizes Social Cognitive Career Theory to a low-socioeconomic-status setting and suggests that strengthening psychological resources and engineering opportunity structures are jointly necessary for translating motivation into demonstrable employability.

## 1 Introduction

Higher education institutions (HEIs) and their function in increasing human resources are crucial to the foundation of national economic development (Choi and Rhee, 2014). In recent years, global trends in higher education have increasingly focused on preparing graduates for the workforce (Moore and Morton, 2015), with universities facing growing pressure to produce employable graduates. Employability has become, and will likely remain, a major concern for various stakeholders in higher education (Bennett, 2018; Holmes, 2013). Employability is also widely recognized as a key indicator of the quality of higher education and a core competency required by the labor market (Abd Majid et al., 2020).

Researchers have extensively studied the concepts of employability. Employability is an individual’s ability to obtain and maintain employment (Harvey, 2001). Beyond this, employability also involves the continuous acquisition of essential work skills to perform tasks effectively and adapt to changes in both internal and external labor markets (Fugate et al., 2004; Vermeulen et al., 2018). In recent years, the concept has expanded to include not only professional and technical skills but also non-technical aspects such as professional identity, personal attributions, and interpersonal relationships (Zegwaard et al., 2017; Bridgstock, 2009). This broader understanding highlights the importance of understanding and improving university graduates’ employability to address evolving labor market challenges.

This study explores employability from the perspective of university students, focusing on the factors they perceive as influencing their employability. Based on qualitative interviews with eleven Chinese university graduates, this research identifies key themes related to employability and presents a framework derived from students’ lived experiences. The findings offer insights into how students interpret employability within the context of their academic and professional transitions, emphasizing their subjective perceptions of what impacts their readiness for the labor market.

### 1.1 Conceptualizing university students’ employability: definitions and determinants

Employability, despite the absence of a single agreed definition, is commonly framed as the ability to obtain, sustain and, when necessary, re-enter employment by mobilizing knowledge, skills, and work-relevant attributes to meet employers’ requirements and achieve professional potential (De Vos et al., 2011; Harvey, 2001).

Substantial studies have examined career readiness, internships, networking, soft skills such as communication and teamwork, and the impact of academic performance on job prospects (e.g., Andrews and Higson, 2008; Soon et al., 2020), as well as how personal attributes, such as motivation and resilience, contribute to securing employment (Lent et al., 1994). External factors including economic cycles and industry demands also shape the context in which graduates search for work (Mason et al., 2009).

These outcomes are ultimately mediated by graduates’ employability, so understanding its composition and development has attracted sustained scholarly attention. Overall, employability formation reflects a blend of internal and educational processes. At the individual level, active engagement in academic activities is crucial, such as high-quality instruction, active teacher-student interaction, and participation in competitions (Pike et al., 2012). Motivation to learn, and the deliberate strategies students adopt for active participation, enhances their ability to internalize knowledge and develop competencies. Beyond core curriculum, participation in humanities courses and extracurricular activities fosters employability by cultivating personal growth and social responsibility (Baek and Cho, 2018), while inspirational teaching and instruction further strengthens students’ motivation, engagement, and learning outcomes (Robinson et al., 2008). Collectively, these engagement processes cultivate higher-order professional competence and social adaptability, capabilities that reinforce employability (Zong et al., 2017).

Building on these processes, internal psychological resources further drive employability development. Self-efficacy, a belief in one’s capability to succeed, is a core determinant of career outcomes (Bandura, 1997) and a critical success indicator of personal employability that can be nurtured (Komarraju et al., 2014; Turner, 2014). Achievement motivation, on the other hand, supports striving for excellence and success (Ye and Hagtvet, 1992), and career ambition encourages goal clarification, sustains persistence, and promotes career transitions (Bandura et al., 2001).

Institutional supports then amplify these internal drivers. Knowledge, skill, and broader competence development are closely related to their teachers (Hennemann and Liefner, 2010). Through program design and aligned teaching, educators bridge academic learning and labor-market expectations, career exploration and planning, ethical values, and work attitudes. This requires university teachers to provide relevant curricula and effective instruction that prepare students for the demands of the job market (Robinson et al., 2008).

Beyond classroom-based provision, experiential pathways supply authentic settings for applying developing competencies. Work-integrated learning opportunities such as internships further strengthen employability. HEIs have increasingly embedded real-work placements and industry projects in their programs to better prepare university students and graduates for the workforce (e.g., Campbell and Zegwaard, 2011; Paryono, 2014).

A wider contextual layer conditions how individual and institutional efforts translate into labor-market advantage. Social background affects various indicators of success (Holmes, 2013). Within that context, social capital, including interpersonal relationships and communication skills, provides visibility and opens doors to career opportunities (Forret and Dougherty, 2004). Against this general framework, the distribution of enabling resources is stratified, motivating closer examination of students from low-income families in the next section.

### 1.2 Employability under socioeconomic constraint: low-income university students factors that influence employability

Access to the enabling factors outlines above is uneven. Low-income or otherwise marginalized students typically possess less social capital and therefore encounter distinctive barriers. Demographic conditions such as race, ethnicity, socioeconomic status (SES), and gender constrain career development (e.g., Brown, 2004; Powers and Wojtkiewicz, 2004). Economically marginalized youth report lower career aspirations for prestigious occupations than their more affluent peers (Howard et al., 2011) and often lack structural and sociopolitical supports needed to secure employment (Mamiseishvili, 2010; Parks-Yancy, 2012).

Research specifically addressing low-income students’ employment outcomes reveals unique challenges faced by this demographic. Financial constraints restrict their participation in internships and networking opportunities, which are critical for accumulating relevant experience and building professional connections (Baker and Henson, 2010). Such pressures can also reorient decision-making toward immediate income, leading low-income students to prioritize short-term or survival employment over positions with stronger developmental trajectories (Buzan and Sheehy‐Skeffington, 2024). Reduced participation narrows mastery experiences, slowing the accumulation of human, social and psychological capital.

These early constraints propagate through academic pathways. Low-income university students face higher risk of withdrawal from universities (Engle and Tinto, 2008) and lower completion rates (Ho and Wei, 2011). Their lower self-confidence and weaker perceived employability relative to peers (Gan and Wang, 2021) further dampen the motivational cycles that typically sustain exploration and skill enhancement. Additional burdens emerge in the form of higher rates of mental-health problems and unmet psychological needs (Wadsworth and Achenbach, 2005), which can erode self-regulatory bandwidth for sustained goal pursuit.

Although studies have identified factors that influence university students’ employability, many lack strong theoretical integration and rely predominantly on descriptive discussion or cross-sectional surveys, offering limited insight into how employability is formed under socioeconomic constraint. To address this gap, this study adopts a qualitative approach to unpack the formative mechanisms shaping employability among university students from low-income families in the Chinese context. It foregrounds optimism as a key component of positive psychological capital and examines how job-search motivation and goal setting foster this capital and, ultimately, enhance employability. In doing so, the study provides an evidence base to refine student support provision and inform employability policy, while advancing understanding of, and practical responses to, the employment challenges confronting low-income university students.

### 1.3 Theoretical background: social cognitive career theory

Bandura’s (1986) reciprocal determinism suggests how individual behaviors arise through interactions between internal thoughts, emotions, and the surrounding environment. Building on this, Lent et al. (1994) developed Social Cognitive Career Theory (SCCT) by integrating key constructs specifically relevant to career development. It posits that personal/cognitive factors, interests, choice goals, and choice actions evolve through reciprocal interactions with contextual supports and barriers.

In SCCT, self-efficacy is closely linked to motivation and engagement in learning, making it a significant predictor of career outcomes (Parker et al., 2006). Together with outcome expectations, self-efficacy informs interests, shapes choice goals, and channels subsequent goal-directed exploration and job-search behaviors (Lent et al., 1994). Contextual supports and barriers (e.g., family resources, teacher guidance, internship access, social capital) affect both the formation of these cognition and the translation of goals into sustained behavior. Thus, under low-income conditions, constrained mastery and modeling opportunities may weaken self-efficacy and dampen adaptive outcome expectations, narrowing employability pathways.

Guided by SCCT, this study explores how internal psychological resources interact with contextual supports and barriers to shape employability among low-income university students.

Specifically, this study aims to address two research questions:

*RQ1*: What are the factors influencing the employability of university students from low-income families?

*RQ2*: What is the mechanism influencing the employability of university students from low-income families?

## 2 Materials and methods

### 2.1 Setting and participants

This study involved 11 graduates from low-income families at an anonymized university in China, selected through counselor/teacher recommendations and convenience access until data saturation was reached. Inclusion criteria were: (1) verified low-income family status (means-tested grants or student loans eligibility); (2) 3–5 year post-graduation; (3) early job acquisition with current job satisfaction; and (4) documented strong employability (dual independent teacher/counselor ratings plus at least two objective indicators such as early offer date, positive internship evaluation, leadership role, scholarship or competition award). Focusing on high-performing low-income cases enabled mechanism tracing.

Table 1 shows that the purposive sample (*n* = 11; 6 female, 5 male) covered multiple discipline areas (engineering, mathematics, chemistry, history, and computer science), now employed in schools, private enterprises, civil aviation, and entrepreneurship. Interviews lasted 49–73 min (*M* = 61.9).

**Table 1:** Interviewees’ information.

No.	Gender	Major	Employment destination	Interview time (min)
A01	Female	Mathematics	School	58
A02	Female	History	School	65
A03	Male	Engineering	Private enterprise	73
A04	Female	Chemistry	School	49
A05	Male	Engineering	Civil aviation	70
A06	Female	Chemistry	School	49
A07	Female	Mathematics	School	59
A08	Male	Engineering	Private enterprise	58
A09	Male	Computer Science	Private enterprise	70
A10	Female	Mathematics	School	70
A11	Male	Engineering	Entrepreneurship	59

### 2.2 Data collection

A semi-structured individual interview protocol was drafted, expert-reviewed and piloted to confirm the general coherence of the framework, then used for individual audio-recorded interviews. The interview focused on the following key questions:

1) What factors or significant events during your university years do you believe have influenced your employability?
2) What internal psychological and external environmental factors do you think affected your employability?
3) How did you overcome the constraints posed by your family’s economic situation?
4) What factors do you think are critical to employability? How did these factors influence you?

### 2.3 Data analysis

Audio recordings were transcribed verbatim and checked against the originals to ensure accuracy. After transcription, the authors reviewed the data multiple times to understand it holistically. A reflexive thematic analysis (RTA) was adopted, with SCCT (Lent et al., 1994) acting as a sensitizing, not prescriptive, framework. Following Braun and Clarke’s (2021) six-phase RTA process, analysis proceeded as:

1) Data familiarization: Transcriptions were read repeatedly to gain an overall sense of the data.
2) Systematic data coding: Three researchers independently coded an initial subset inductively (to capture new meanings) and deductively (attending to SCCT constructs), then discussed and aligned coding practices.
3) Generating initial themes: Codes were clustered into provisional, meaning-rich themes with clear organizing concepts.
4) Developing and reviewing themes: Candidate themes were iteratively checked against the full dataset; boundaries and relationships were refined in team meetings.
5) Refining, defining, and naming themes: Final labels emphasized the central meaning of each theme and its relation to others.
6) Writing the report (Producing the analytic narrative and theme maps): Researchers produced the analytic narrative and visualized theme relationships.

Rather than treating “saturation” as a numeric endpoint, we used an information power logic (Malterud et al., 2016): coding ceased when additional interviews no longer added conceptual nuance to existing themes. Reflexive memos documented analytic decisions and researcher positioning to support transparency and rigor (Braun and Clarke, 2021).

When developing themes, we attended to patterns of recurrence (e.g., how widely a code appeared across participants), but did not treat frequency as a proxy for importance. In line with RTA, themes were judged primarily on their conceptual significance and relevance to the research question, rather than on numerical prevalence alone (Braun and Clarke, 2021).

### 2.4 Ethics approval statement

Ethical approval for this study was obtained from the Science and Technology Ethics Committee of Jilin Normal University (approval number: KJLL20250404). Written informed consent was obtained from all participants prior to data collection, including consent for participation and for the publication, and all interviews were conducted in a confidential setting.

## 3 Results

### 3.1 Positive psychological capital for job search

Across all cases, positive psychological capital, principally self-efficacy and optimism, functioned as a foundational mechanism converting disadvantage into proactive job-search preparation.

#### 3.1.1 General self-efficacy

Participants framed prior mastery and role-taking experiences (student teams, competitions, internships) as evidence that they could “bridge” structural gaps (e.g., weaker family connections), reinforcing a generalized belief in capability. One graduate summarised, “I have confidence that I can succeed in entrepreneurship … I formed small teams and student societies, coordinated and ran them … and that experience showed me I can do it” (A11).

#### 3.1.2 Self-reliance

A strong sense of *self-reliance*, that is, striving on one’s own, was also displayed throughout the interviews. “Coming from a rural family … knowing my parents had no connections, I studied even harder, with much effort” (A01). Such structural scarcity (rural background, lack of connections) was translated into intensified personal effort.

#### 3.1.3 Optimism

*Optimism* appeared in all participants’ accounts and operated as a resilience lens: difficulties were reinterpreted as developmental prompts rather than deterrents. “Take the challenge as a good thing, and going through these things makes me calmer and more mature” (A08). Another participant emphasized affective regulation: “I have always maintained a positive and upbeat outlook” (A02). This optimistic framing sustained effort during demanding scholarship applications and early job searches, buffering perceived deficits in social capital.

Mechanistically, self-efficacy and optimism jointly reinforced achievement expectations and reduced avoidance cognition, supplying the cognitive-emotional platform for subsequent motivational drives.

### 3.2 Motivation for job-seeking action

Building on this psychological capital, participants articulated a layered motivational complex comprising achievement motivation, proactive job-seeking initiative, and a responsibility-based persistence grounded in both family obligations and reciprocity for family support.

#### 3.2.1 Achievement motivation

Achievement motivation appeared as a readiness to iteratively test opportunities despite uncertainty: “If I have a chance to look for a job, I will try. If I fail, it doesn’t matter, I just keep trying.” (A05). This cycle of attempt–reflection–re-attempt turned setbacks into feedback rather than final verdicts, sustaining forward momentum.

#### 3.2.2 Job-seeking initiative

Early labor-market analysis and strategic action. When family networks offered little leverage, several students described going far beyond “sending more CVs”. This includes conducting their own early analysis of the labor market by scanning recruitment information, asking insiders about required skills and attending job fairs ahead of time, and using this knowledge to guide selective skill upgrading and network use as substitutes for family capital. A08 exemplified this by systematically tapping into the alumni network in place of family social capital: he “contacted seniors from several cohorts, asked which skills their jobs needed, and set myself a timetable to learn circuit design and programming language so I could choose between electronics or software engineering.” Here, early reading of labor-market demands, filtered through seniors’ experience, directly shaped which competencies he invested in. A10 likewise acted ahead of the recruitment calendar and used experiential learning to build a tangible portfolio: “As soon as a job-fair notice went online we caught the first bus there; meanwhile I drilled demo lessons and took weekend jobs so I’d have something concrete to show if an opportunity came.” In contexts where parents could not offer internship placements or insider contacts, early market scanning, targeted upskilling and network tapping collectively functioned as replacements for familial capital, turning structural scarcity into a self-directed competitive edge.

#### 3.2.3 Sense of responsibility

*Sense of responsibility* toward themselves and their families primarily manifested as a situated reinterpretation of their economic circumstances. This is a commitment to alleviate the family’s financial burden and to shoulder household economic responsibilities. This sense of responsibility reframed financial assistance as a trigger for future labor-market preparation: “A scholarship that repaid my loan and eased my family’s burden pushed me to study harder and build abilities employers need” (A01). Here, perceived improvements in the family’s economic prospects reinforce self-efficacy and sustain structured competence accumulation geared toward easing parents’ financial pressure and contributing to household expenses, directly feeding employability development.

Such responsibility-based persistence encompassed both practical duties (such as easing the family’s financial burden and preparing to support parents in the future) and a sense of reciprocity for the support received during university. It operated as a conversion mechanism: family-oriented outcome expectations stabilized elevated goals and energized sustained competence and search behaviors.

### 3.3 Goal-oriented job-search actions

#### 3.3.1 Career aspirations

Participants first voiced broad *career aspirations* that gave a directional vision of future development. They described a desire to enter economically developed cities to access wider platforms, richer resources and broader networks. One participant noted: “A lot of people … go to the outside … I also think of working in big cities after graduation to see the wider world” (A08). These forward looking statements sustained motivational energy but remained, for some, at a general directional level.

#### 3.3.2 Clear career goals

A second layer was the articulation of *clear career goals.* Participants moved from broad aspirations to structured sequences: first consolidating core technical competence, then pursuing depth and positioning for future leadership. As A09 stated: “[M]y goal right now is to nail software and become a leader in that field.” Where goals reached this level of specificity, participants reallocated limited time and financial resources toward high-leverage actions: targeted advanced modules, project- or sector-aligned internships, selective competition or certification efforts, early résumé tailoring and senior consultation.

Geographic aims (entering developed cities) were thus coupled to concrete capability and signaling steps rather than remaining as diffuse hopes. Direction supplied purpose; clarity supplied procedure. This translation from vision to sequenced, resource-aware steps converted psychological capital into deliberate competence accumulation and earlier labor-market signaling, accelerating employability formation and differentiating participants who articulated staged milestones from those who retained only a directional aspiration.

### 3.4 Extrinsic factors at home and school

External conditions at home and in the university environment shaped employability formation, while simultaneously catalyzing distinctive internal responses, namely motivation to study harder and self-reliance.

#### 3.4.1 Family economic status

Financial constraint and limited social capital formed the structural backdrop shaping access to resources. One participant recalled: “I entered university through the ‘green channel’ with student loans. At first, I did not know about scholarships.” (A01). This absence of early knowledge curtailed perceived options and heightened the salience of discovering funding channels. As noted earlier by A01 regarding repaying student loan through a scholarship, the later scholarship was not only financial relief but a conversion event: after repaying, it freed cognitive bandwidth, and provided a visible competence signal. Crucially, the experience amplified a *motivation to study harder* and affirmed *self-reliance*, meaning self-directed, agentic effort in the absence of familial capital, not generic diligence but a sharpened, responsibility-coloured intention to secure subsequent scholarships and maintain a performance profile convertible into employability capital.

Here, “study harder” did not mean diffuse diligence; it denoted selective effort allocation (maintaining grade thresholds, sequencing further scholarships, consolidating core skills) enacted through autonomous information search and application strategy. Thus low family economic status operated less as a static deficit and more as a catalyst for strategic information seeking, self-directed striving that preceded external support, and signalling behavior. Internal drive is reframed as instrumental redeployment, transforming scarcity into human and reputational capital.

#### 3.4.2 University prestige-linked job-search barriers (external gate-keeping)

Participants described a stratified hiring environment in which institutional prestige functioned as a first-pass filter: employers “prioritise” higher-status universities, raising perceived thresholds for applicants from “ordinary” institutions. As one noted: “Graduates from ordinary universities face greater challenges … many employers prioritize graduates from top universities … and don’t even consider us. I cannot give up. I adjusted and worked on improving myself to apply for another job.” (A04).

The contextual barrier and status-based exclusion elicited not withdrawal but *determination to be strong* and iterative, self-directed adjustment. Thus prestige hierarchy operated as a motivational discriminator: it sharpened appraisal of skill gaps and channeled effort into compensatory competence building rather than diffuse effort. Mechanistically, this reflects an SCCT pathway where a contextual barrier reinforces resilient self-efficacy beliefs and choice goal maintenance, sustaining adaptive choice actions (selective upskilling, strategic re-applying) that preserve and gradually enhance employability signals despite structural filtering.

#### 3.4.3 Teachers’ active guidance

Accessible, task-focused teacher support converted diffuse motivation into structured academic and career planning. Participants contrasted reduced parental oversight at university with timely pedagogical intervention that maintained study routines and sharpened goal specificity. “[M]y teachers were very helpful in guiding my research direction and career plans … Whenever I needed help, they were dedicated to offering support” (A02). Guidance thus reinforced self-efficacy and efficient allocation of effort to competence-building activities.

#### 3.4.4 Participation in extracurricular activities

Student societies and competitions functioned as low-risk arenas to practise and publicly signal transferable skills, including communication, organization, and leadership. “Participating in social activities is important … I use these opportunities to improve myself across different aspects” (A02). After placing in a speech contest, one student “felt much more confident about presenting in public” (A01), while another joined mixed-major clubs “to meet different people and practise my communication skills” (A04). Such intentional participation framed extracurricular space as structured skill rehearsal and incremental signaling, strengthening presentation, interpersonal and coordination self-efficacy.

#### 3.4.5 Experience transfer from seniors

Seniors’ narratives offered vicarious learning about timelines, evaluation criteria and pitfalls, reducing exploratory drift and accelerating goal setting. As A05 put it, “[s]eniors shared their experiences, and after listening to them, I realized I couldn’t just drift. I needed to set career goals early and plan accordingly to improve my abilities at university.” Hearing concrete accounts of how seniors had navigated courses, internships and competitions made such trajectories feel attainable, thereby strengthening students’ beliefs that they could reach similar standards. In terms of self-efficacy, these senior peers provided vicarious experiences that bolstered self-efficacy, complementing the confidence gained from extracurricular participation and often proving more impactful than formal teacher instruction because their stories were perceived as more relatable and directly applicable.

#### 3.4.6 Internships and part-time work experiences

Work placements supplied authentic performance feedback, revealing theory-practice gaps and directing targeted upskilling. “During the internship, I saw how little practical experience I had compared to what I learned in theory. Afterwards, I worked harder … on hands-on practice.” (A07). Appraisal of the gap prompted targeted strengthening of applied techniques and workflow familiarity, which in turn accelerated the emergence of demonstrable experience as a labor-market signal.

Collectively, these external, institutional supports and experiential arenas transformed psychological capital (Sections 4.1–4.3) into selective competence accumulation and signaling behaviors via three linked processes: (1) informational structuring (teachers, seniors), (2) confidence and soft-skill signaling (extracurricular), and (3) performance feedback loops (internships). This alignment advanced employability despite initial structural constraints.

### 3.5 Competencies required for job seeking

Participants framed job-seeking ability as a constellation of competencies that employers notice, including learning and self-management, resilience and positive attitude, and socio-communicative/self-regulatory skills, and they viewed developing these capacities as essential for standing out in a competitive labor market.

#### 3.5.1 Learning and self-management ability

Participants framed disciplined learning and time orchestration as the internal infrastructure that converts motivation into cumulative capability. As A03 expressed: “The main thing during university is to develop your ability to learn; it matters for your future career.” Self-management sustained selective upskilling and reduced exploratory drift by “reasonably balance your time in study and life and ensures that all aspects are well-developed” (A08), thus reinforcing prior goal clarity.

#### 3.5.2 Resilience and positive attitude when facing adversity

Economic strain and prestige filtering were reinterpreted as challenges to be neutralized rather than determinants. Graduates still believed that external environmental factors are not decisive in job hunting. Instead, maintaining a proactive approach to overcome difficulties and continuously improving their employability is essential to job-search success: “Do not feel insecure if you come from an ordinary school or university. I initially struggled too but became proactive in preparing for job applications. External factors exist, but the key is … whether your abilities meet the job requirements.” (A03). This appraisal style anchored persistence, protected self-efficacy from rejection cycles and maintained effort on competence accumulation rather than rumination.

#### 3.5.3 Integrated self-regulation and socio-communicative capital

Participants shifted from teacher-driven study autonomous regulation: “At university no one keeps telling you to study… I pushed myself by joining activities to train, especially speeches and hosting, using those platforms to practice and show myself.” (A04). Activity participation here was not treated as an end but as a deliberate rehearsal environment producing composure, audience-facing confidence and refined verbal delivery. A reflective correction cycle emerged: initial academic relaxation was recognized and adjusted: “I relaxed at first… then realized the problem and began to study again while still taking part in activities I enjoy.” (A04). Mechanistically, autonomous regulation, including scheduling and reflective adjustment, and self-presentation practice co-evolved, expanding a transferable portfolio, such as communication poise, opportunity utilization, and self-monitoring, that enhances interview readiness and signaling efficiency.

### 3.6 Employability development “through” and “as” adaptive practice

#### 3.6.1 Cross-case themes

Reflexive thematic analysis in this study generated four higher-order themes capturing patterned meanings in participants’ accounts of employability. Two speak to internal psychological resources and agency, and two to the direction and enactment of that agency. All are framed by external contexts.

Theme 1: Psychological capital as a springboard. Participants repeatedly invoked self-efficacy and optimism as the felt resources that enabled them to “catch up” despite financial hardship. Initial low self-esteem linked to poverty was often reframed as motivation.

Theme 2: Resource-compensatory career proactivity. Job-seeking initiative, self-reliance, determination and responsibility surfaced as strategic behaviors, including early information scanning, tailoring materials, consulting seniors, triggered by recognizing limited familial networks.

Theme 3: Goal clarity as compass. Career aspirations and clear goals channeled motivation into concrete planning, consistent with evidence that aspirations orient later achievement.

Theme 4: Capability enactment through adaptive practice. Learning/self-management, resilience, and socio-communicative/self-regulatory capacities were described as abilities honed through internships, extracurricular roles and iterative practice.

#### 3.6.2 Across-case synthesis

**Figure 1:**
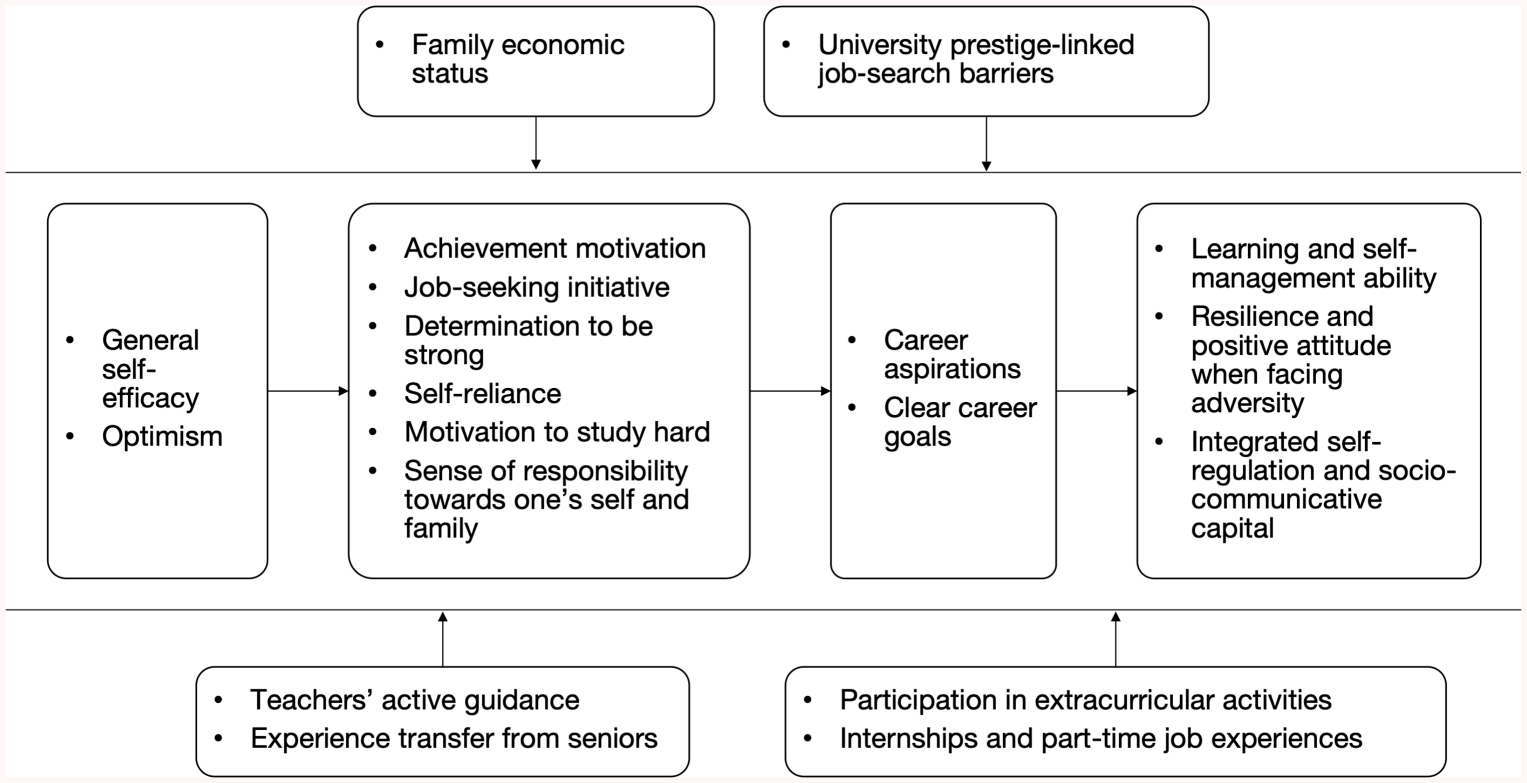
Theme map of factors influencing the employability of university students from low-income families.

Moving across cases, 19 sub-themes were collated, as shown in Figure 1, and clustered into the four themes above (psychological capital → motivational drives/proactivity → goals → abilities/skills), all encircled by family and university contexts. Section 5.2 unpacks how these blocks interact sequentially and recursively, and integrates them into a conceptual model.

## 4 Discussion

### 4.1 Intrinsic and external factors affecting the employability of university students from low-income families

Drawing on Lent et al.’s (1994) SCCT, the analysis positions employability at the nexus of personal agency and structural context for university students from low-income Chinese families. Prior studies have highlighted both internal resources (such as graduates’ competencies and career-related aspirations) and external conditions (such as institutional strategies, labor-market demand and skills policy) in shaping employment outcomes (e.g., Ahmed et al., 2015; Blázquez et al., 2018; Cacciolatti et al., 2017). Evidence from Latin American contexts likewise highlights how employers’ interpretations of educational credentials, soft-skill gaps, and the structural configuration of labor markets shape university students’ employment chances (e.g., Nogales et al., 2020; Moya Loaiza et al., 2025). Building on this literature, our findings further illustrate how psychological capital, resource-compensatory proactivity and goal clarity interact with such contextual constraints and supports in shaping perceived employability.

Psychological capital (Theme 1) and proactive striving (Theme 2) align with findings that self-efficacy and achievement motivation underpin employability (e.g., Komarraju et al., 2014; Turner, 2014). Participants initially had low self-esteem and confidence tied to financial hardship, impacting their motivation for career development (Alivernini et al., 2023). *Optimism* supplied an affective buffer for sustaining persistence in the face of difficulties, aligning with Seligman’s (2002) explanatory style theory of optimism. Goal clarity (Theme 3) channeled these drives, making participants determined, upwardly mobile, and driven to succeed (Ćurić Dražić et al., 2018). Consistent with Bandura et al. (2001), participants’ articulated career aspirations and concrete study plans oriented subsequent achievement behaviors.

Less commonly articulated, but still salient, traits (e.g., strong job-seeking initiative, self-reliance, determination to be strong, sense of responsibility) appear idiosyncratic to individuals yet meaningful for employability. Their low visibility may reflect genuine heterogeneity or simply the limits of single-interview depth.

On the contextual side, family finances, institutional capital, and opportunity structures mattered. As Holmes (2013) found, social background, structures the “field” of possible moves; likewise, our low-SES participants described constrained access to elite programs, employer networks, and recruitment channels, echoing that poor SES impede career development (Howard et al., 2011; Powers and Wojtkiewicz, 2004). Yet the participants’ responses show how compensatory resources operate: targeted courses and proactive faculty guidance acted as verbal persuasion and opportunity scaffolds in SCCT terms, supporting self-efficacy and sustaining goal pursuit (Ahmed et al., 2015; Baek and Cho, 2018; Blázquez et al., 2018; Robinson et al., 2008). Internships and part-time work experiences then converted intentions into demonstrable skills and networks (Campbell and Zegwaard, 2011; Paryono, 2014).

**Figure 2:**
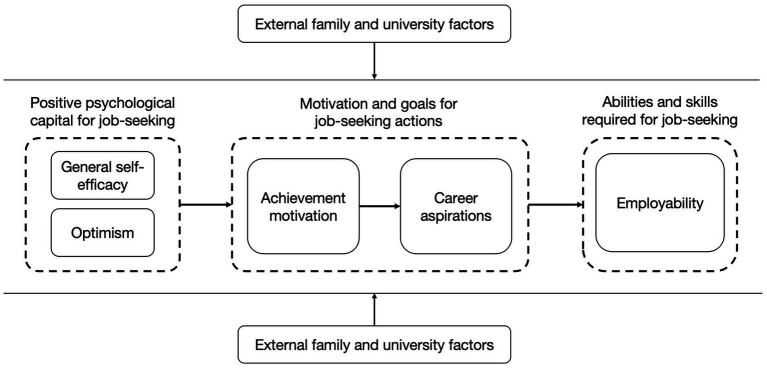
Integrated conceptual model of employability development among university students from low-income families.

These influences do not operate in isolation; they interact sequentially and recursively. The section below develops this analytic narrative and visualizes it in Figure 2.

### 4.2 An integrated analytic narrative and conceptual model of factors influencing employability

This study extends domestic theorizing and qualitative work on university students’ employability, particularly for those from low-income families, by moving beyond generic definitions and structures (e.g., Fugate et al., 2004), correlational focus on employment outcomes (e.g., Andrews and Higson, 2008; de Schepper et al., 2021; Weber et al., 2013), or determinants (e.g., Harvey, 2001; Tomlinson, 2012). In a Chinese context where social and institutional conditions differ, the findings of this study demonstrate the need to localize employability and to unpack its psychosocial mechanism for students with limited financial and career support.

Guided by SCCT but driven by data, we interpret a sequential yet recursive storyline:

1) Positive psychological capital ignites action. General self-efficacy and optimism provide initial momentum.
2) Proactivity sustains effort. This momentum energizes achievement motivation, job-seeking initiative, determination to be strong, self-reliance, motivation to study hard, and a sense of responsibility, producing sustained effort.
3) Aspirations and goals give direction. Motivation crystallizes into career aims and concrete plans.
4) Abilities are enacted and reinforced. Goal-directed behaviors cultivate learning/self-management, resilience and socio-communicative/self-regulatory competence, thereby strengthening employability.

External conditions include family economic status, prestige-based university stratification, teachers’ guidance, seniors’ experience transfer, extracurricular participation, internships and part-time work. These contextual factors shape each link in the chain as filters (limiting access) and scaffolds (converting intention into competence). Crucially, the process loops back: mastery experiences feedback to reinforce self-efficacy (Chemers et al., 2001; Torres and Solberg, 2001), sustain optimism (Seligman, 2002), and elevate subsequent goals. In SCCT (Lent et al., 1994) terms, this study shows how self-efficacy, outcome expectations, and goal beliefs interact dynamically rather than linearly. Overall, cultivating self-efficacy and optimism is pivotal for low-income students, but these internal resources must be paired with opportunity structures to translate motivation into demonstrable employability.

Drawing together the analysis of factors and this analytic narrative, Figure 2 presents an integrated conceptual model. Grounded in our data, it aligns with SCCT’s core tenets (Lent et al., 1994) and Bandura’s (1986) emphasis on motivation–behavior linkages, yet specifies how positive psychological capital and contextual scaffolds operate sequentially and recursively for low-SES Chinese students. Prior employability accounts have often privileged either individual attributes or structural constraints in isolation (e.g., Harvey, 2001; Tomlinson, 2012). By contrast, our model explicitly links psychological capital, motivational processes, and contextual supports, illustrating how they interact rather than treating them as separate explanations.

The model in Figure 2 comprises three linked blocks:

1) Left block: Positive psychological capital (self-efficacy, optimism) provides the motivational “fuel”;
2) Center block: Motivation and goals (achievement motivation → job-seeking initiative/self-reliance/responsibility → career aspirations and clear goals) translate that fuel into direction;
3) Right block: Employability abilities/skills (learning and self-management, resilience, socio-communicative/self-regulatory capital) represent enacted capability.

Family and university contexts frame these blocks from above and below, functioning as constraints and resource platforms that modulate each transition. The process is sequential yet dynamic, with successful experiences progressively strengthening psychological capital and resetting the cycle at a higher level. Hence the dual imperative is clear: build internal psychological resources and construct opportunity structures where those resources can be exercised and evidenced.

Although our model depicts general self-efficacy and optimism as antecedents of achievement motivation and job aspirations, this directional ordering should be interpreted with some caution. Within the SCCT framework that guides our study, self-efficacy and related positive expectancies are typically conceptualized as key motivational resources that precede goal setting and shape subsequent behavior (Lent et al., 1994). Our qualitative accounts were largely consistent with this view: many students narrated increases in self-belief and optimism as the starting point that energized achievement motivation and proactive job-seeking, which then crystallized into clearer aspirations and plans. At the same time, achievement goal research often positions goals and aspirations themselves as antecedents, with self-efficacy sometimes acting as a mediator between goals and outcomes (e.g., Elliot and McGregor, 2001). However, our narratives did not provide clear evidence for this alternative ordering; rather, they predominantly showed increases in self-efficacy and optimism preceding and shaping students’ aspirations and goals. Consistent with SCCT’s emphasis on reciprocal influences, the arrows in Figure 2 should therefore be read as indicating the dominant pattern observed in this sample rather than definitive causal pathways. Disentangling alternative orderings, such as goals → self-efficacy → employability versus self-efficacy → goals → employability, will require longitudinal or structural modeling in future quantitative research.

It is also worth noting that, although this pattern did not result from an intentional sampling strategy, the mechanisms described here reflect pathways observed among low-income students who had persisted in higher education and remained engaged with institutional support, rather than capturing the full range of trajectories within this population.

## 5 Conclusion and implications

To examine the influences that shape and enhance employability among university students from low-income families, guided by SCCT, this study analyzed 11 interviews using reflexive thematic analysis. By synthesizing 19 coded themes, this study constructed a data-driven conceptual model that clarifies how multiple factors combine to shape these students’ employability.

Drawing on Lent et al.’s (1994) Social Cognitive Career Theory (SCCT), it was concluded that key factors such as general self-efficacy, optimism, achievement motivation, and career ambition are critical. Establishing clear job-seeking goals further strengthens career aspirations and improves employability, forming a structured pathway for intervention strategies. This study focuses on identifying and describing the pathways through which key variables influence employability within the SCCT framework, rather than precisely testing their mediating or causal relationships. Such fine-grained mechanistic examinations are better suited to subsequent quantitative research, which we intend to pursue in future work.

Self-efficacy belief, a core SCCT construct, is central to this model. High self-efficacy enhances confidence, nurtures belief in workplace success and drives goal pursuit. Optimism, another vital psychological trait, fosters resilience, enabling students to maintain a positive outlook, perceive opportunities, and confront obstacles proactively, thereby improving competitiveness. Accordingly, career guidance for low-income students should prioritize boosting self-efficacy and nurturing an optimistic mindset. In addition, enhancing achievement motivation and career aspirations is vital: striving for success in job seeking operates as an intrinsic motivator that catalyzes employability development. Importantly, this study provides clear guidance for integrating these findings into career counseling models. Specifically, SCCT-informed counseling frameworks should be designed to intentionally target and cultivate these key psychological factors. Interventions can be structured to progressively assess and strengthen these variables, thereby translating the conceptual understanding of employability development into a practical, psychology-informed counseling process aimed at empowering students. In practical terms, such career counseling models could: (1) begin with systematic assessment of students’ self-efficacy, optimism and achievement motivation; (2) incorporate activities that build mastery experiences and vicarious learning (e.g., structured peer mentoring with seniors, guided reflection on past successes); and (3) embed structured goal-setting and action-planning components that translate aspirations into concrete job-search behaviors and skill-building plans.

### 5.1 Research limitations and future prospects

Nevertheless, this study has limitations. First, the small and specific sample may limit the generalizability of the findings and the proposed model. The participants were all Chinese university students from low-income families, and many themes, such as strong senses of responsibility, hard work, and self-reliance, are embedded in this cultural and socio-economic context. As such, the model should be viewed primarily as context-sensitive rather than universally applicable, and its relevance in other cultural settings and among students from different social classes will need to be examined in future research.

Second, the qualitative design offers depth but yields primarily theoretical insights with limited empirical validation. Future work could therefore design and evaluate targeted intervention programs derived from this model, testing its applicability across institutions and regions, for example through mixed-method or longitudinal evaluations of SCCT-informed counseling modules. Such work would move beyond conceptual development to assess when and for whom model-based counseling can most effectively support low-income students to develop their employability and improve their longer-term career outcomes. Complementary quantitative studies with larger samples could also estimate the relative strength of different factors identified here, providing the kind of numerical weighting of influences on employability that lies beyond the scope of the present RTA-based analysis.

Third, while our analysis has foregrounded how some low-income students actively reframed barriers as challenges and mobilized resources to enhance their employability, it is important to acknowledge whose experiences remain less visible in this account. The participants were all students who had persisted in higher education and were willing to engage in an interview about their careers. Many of them were relatively proactive and had at least partial access to institutional supports, such as scholarships or career services. As a result, the study primarily illuminates mechanisms that enable a subset of low-income students to convert constraints into resources, rather than mapping the full distribution of outcomes within this population. It is plausible that a larger group of low-income graduates struggle to overcome structural barriers and face persistent underemployment or exclusion; their trajectories are under-represented in our data and require further investigation. Future research should purposively include low-income students who have dropped out of higher education, experienced prolonged unemployment, or disengaged from career support, in order to examine how the absence of psychological capital and contextual scaffolds constrains employability trajectories.

Additionally, some participants described status-based disadvantages in recruitment, for instance, feeling that graduates from “ordinary” universities were filtered out or overlooked. However, they rarely articulated explicit critiques of status-based exclusion or questioned the quality of “normal” higher education. Instead, they tended to frame disadvantage in terms of personal effort and the need for additional support. While this absence of structural critique is itself revealing, our interview schedule did not systematically probe how students make sense of educational stratification and status hierarchies. As a result, our data do not allow a deeper analysis of how beliefs about mainstream higher education shape employability, which we therefore identify as an important direction for future research.

## Data Availability

The raw data supporting the conclusions of this article will be made available by the authors, without undue reservation.
